# Comparison of Hemodynamic Response following Spinal Anesthesia between Controlled Hypertensive and Normotensive Patients Undergoing Surgery below the Umbilicus: An Observational Prospective Cohort Study

**DOI:** 10.1155/2021/8891252

**Published:** 2021-07-13

**Authors:** Leake Gebrargs, Bereket Gebremeskel, Bacha Aberra, Assefa Hika, Yusuf Yimer, Misrak Weldeyohannes, Suleiman Jemal, Degena Behrey, Abere Tilahun

**Affiliations:** ^1^Department of Anesthesiology, College of Health Sciences, Aksum University, Axum, Ethiopia; ^2^Department of Anesthesiology, College of Health Sciences, Addis Ababa University, Addis Ababa, Ethiopia; ^3^Department of Adult Health Nursing, College of Health Sciences, Aksum University, Axum, Ethiopia; ^4^Department of Anesthesiology, College of Medicine and Health Sciences, Adigrat University, Adigrat, Ethiopia

## Abstract

**Background:**

Hypotension and bradycardia are the most common complications associated with spinal anesthesia and more common in patients with a history of hypertension. Regular use of antihypertensive medications can prevent these complications. The occurrence of hypotension under spinal anesthesia among controlled hypertensive and normotensive patients with age 40 years and above is still debated. The objective of the study was to compare blood pressure and heart rate changes following spinal anesthesia between controlled hypertensive and normotensive patients undergoing surgery below the umbilicus at Black lion hospital, Addis Ababa, Ethiopia, 2020.

**Method:**

A hospital-based prospective cohort study was conducted. A total of 110 elective patients with controlled hypertension (55) and normotensive (55) patients who underwent surgery with spinal anesthesia at black lion hospital during the study period were included. The sample was selected using a systematic random sampling technique. Continuous data of independent and dependent variables were analyzed using an independent sample *t*-test for normally distributed and Mann–Whitney *U*-test for nonnormally distributed between the study groups. Categorical variables between the study groups were analyzed using the chi-square test. Descriptive data were displayed using tables and figures. For continuous and categorical variables, a *p* value <0.05 was considered statistically significant.

**Results:**

The incidence of hypotension in the controlled hypertension group (23.6%) was higher than the normotensive group (7.3%) with *p* value of 0.018. The occurrence of bradycardia was seen to be 12.7% in each group with a *p* value >0.05. There was a statistically significant difference in the mean systolic blood pressure, mean arterial pressure, mean heart rate, and vasopressor consumption at the measurement time interval between controlled hypertension and normotensive groups.

**Conclusion:**

Under spinal anesthesia, patients with controlled hypertension are more likely to develop hypotension than normotensive patients, but on the occurrence of bradycardia, there was no statistically significant difference between the two groups.

## 1. Introduction

The hemodynamic instability after spinal anesthesia is common intraoperatively and the incidence of intraoperative hypotension ranges from 8 to 33% depending on the parameters used to define it (systolic blood pressure, usually <80–90 mmHg) or on a 25%–30% reduction of the initial systolic blood pressure [1].

Risk factors for early hypotension and bradycardia after spinal anesthesia in nonobstetrical populations include a block height ≥T5, age ≥40 years, female gender, weight, height, body mass index >30 kg/m^2^, ASA physical status II and above, history of hypertension, history of antihypertensive therapy, in case of ongoing beta-blockers therapy, diabetes mellitus, anemia, lower baseline heart rate <60 beats/minute, baseline systolic blood pressure (SBP) < 100 mmHg, and spinal puncture above the level of L3 > L4 [2, 3]. High-level blockade (≥T5) and the age ≥40 years) are the two main predicators of hemodynamic instability and hypotensive complication after spinal anesthesia, which has an incidence of 15.3 to 33% [4–6].

The current practice to treat hypotension is systolic BP of less than 85 or 90 mmHg or a decrease of greater than 25–30% from baseline. However, the hemodynamic change underlies the decrease in SBP has not been investigated fully [7]. Various techniques are being used to prevent hypotension and bradycardia which include coloading IV fluid, atropine, vasopressor, and physical methods such as table down and patient head down [8].

The primary outcome of the present study is to determine the percentage change in systolic blood pressure and heart rate after spinal anesthesia among controlled hypertension and normotensive patients who underwent surgery in this institution as the techniques of monitoring, the types of anesthetic and antihypertensive medications, and the management protocols used may vary in our context.

In addition to this, controversial results were found about the incidence of hypotension in different literature. Some studies showed no significant difference between the normotensive and controlled hypertension patients in the incidence of hypotension caused by spinal anesthesia with hyper basic bupivacaine at a *p* value >0.05 [2, 3]. In another study, hypotension due to spinal anesthesia was approximately twice as common in controlled hypertension patients [9, 10]. In both studies, the authors conducted a study to evaluate and compare the hemodynamic alterations in both normotensive and controlled hypertensive patients under spinal anesthesia. The findings of this research will not only address the above controversies but also help anesthesia professionals to predict the hemodynamic responses after spinal anesthesia in these patient groups to provide safe and effective anesthesia using appropriate medications, techniques, and monitoring.

## 2. Methods

### 2.1. Study Objective

This study aimed to compare the change in blood pressure and heart rate following spinal anesthesia between controlled hypertension and normotensive patients undergoing surgery below the umbilicus.

### 2.2. Study Design

An observational prospective cohort study was employed from October 30, 2019–January 30, 2020, after ethical approval (no: 9/2019, Dec 16, 2019) was obtained from the Addis Ababa University Ethical committee.

### 2.3. Study Setting

The study was conducted at Black Lion Hospital (BLH) in urology, orthopedic, gynecological, and general surgery operation theater. Black lion hospital is the largest federal government hospital in the country and it is under the management of Addis Ababa University College of Healthy Science. The hospital has about 800 beds and 14 operation theatres, out of which the two operation theaters are for urology and general surgery, two rooms for elective orthopedics, and one room for gynecological surgery procedures. Approximately, 7000–9000 patients undergo surgery each year.

### 2.4. Study Participants

All ASA I and II patients with a heart rate between 60 and 100 bpm (controlled hypertension and normotensive patients), BMI <30 kg/m^2^ [11, 12], and an age of 40 years and above who were presented for elective surgery below the umbilicus under spinal anesthesia and those who fulfill inclusion criteria were included in the study. Pregnant mothers; patients who had blood loss necessitating transfusion; patients with chronic hypertension, diabetes mellitus, known cardiac disease, renal disease, and coagulopathy; patients with contraindication to subarachnoid block, patients with known sensitivity to the study drugs; and patients refusing either regional anesthesia or the study technique were also excluded from the study. The combination of spinal blocks with other types of anesthesia, partial spinal blocks, and total spinal were also excluded.

### 2.5. Study Variables

In this study, the independent variables were sociodemographic and operative data (age, sex, height, weight, BMI, etc.), other exposure variables (preoperative antihypertensive and anxiolytic medications, spinal anesthesia, fluid coload (ml), time of LA administered into CSF (in seconds), duration of surgery, and blood loss), and peak level of sensory block. The dependent variables were hemodynamic changes (changes in blood pressure and heart rate).

### 2.6. Determination of Sample Size and Sampling Techniques

#### 2.6.1. Determination of Sample Size

The sample size was determined using Epi Info 7 statistical calculator for an independent cohort study. The following assumptions were considered to estimate the sample size required for the study. A power of 80%, confidence interval 95%, and ratio of normotensive to controlled hypertension patients were 1 : 1. In a previous study done in Pakistan [13], the incidence of hypotension in a normotensive patient was 34% and the incidence of hypotension in controlled hypertension was 62%; a sample size of 50 patients was needed per group. Adding 10% for nonresponse, the total sample size was 110 (55 subjects to each group).

#### 2.6.2. Determination of Sampling Technique

The daily operation schedule list from operation theaters in orthopedic, urology, general, and gynecological surgery was used as a sampling frame. The number of participants selected from each operation theater was based on population proportion allocation after the patients were categorized into four specialty groups. Accordingly, from the total of 292 elective cases which were operated at black lion hospital under spinal anesthesia in the last three months period, urological (84), orthopedic (112), gynecological (48), and general surgery (48) patients obtained from the registration logbook were used to determine the sample size of the study participants in the current study. Participants were selected using the systematic sampling technique. So, sampling interval (*k*) was calculated for each specialty as *K* = *N*/*n*, where *N* = total study population, *n* = total sample size, and the sampling interval was 3 in all specialty groups. The first participant was selected randomly using the lottery method. Then, *K* = 3 patients were included in this study from the daily operation schedule list until the required sample size was met and they were grouped based on whether they were normotensive or controlled hypertensive patients. Therefore, the total 110 study participants were finally taken from each specialty with the following ratio: urological (32), orthopedic (42), gynecological (18), and general surgery (18). We spent two extra weeks to reach the required number of patients from each specialty as stated above and to get an equal sample size in both normotensive and controlled groups. Patients were counseled during preoperative examination and were included in the study after obtaining informed consent from each of them in their own language.

### 2.7. Conduction of Anesthesia

All antihypertensives were continued until the day of surgery except for diuretics, ACEI, and long-acting calcium channel blockers. All hypertensive patients' group received 0.15–0.2 mg/kg of diazepam in the morning of the surgery and nifedipine 20–30 mg PO the night before surgery. After receiving the patient in the operating room, documents were checked, a brief clinical examination was done, and standard ASA monitors were attached including pulse oximetry, ECG, NIBP, and temperature probe. Baseline heart rate (HR), systolic blood pressure (SBP), diastolic blood pressure (DBP), and mean arterial pressure (MAP) were recorded before the administration of spinal anesthesia. After establishing IV access with an 18G cannula, an average of 15 ml/kg crystalloid solution was administered as fluid coload to prevent intraoperative hypotension, and patients were followed for one hour after spinal anesthesia. After proper aseptic precaution, spinal subarachnoid block (SAB) was performed in the sitting position, at L3-L4 intervertebral space through a midline approach by a 22G Qunicke type spinal needle and 3 ml of 0.5% hyperbaric bupivacaine was administered after confirming needle location. Patients were placed supine with a pillow under their heads. All patients received the calculated crystalloid infusion during the procedure. Surgery was allowed after adequate sensory and motor block was confirmed. In the postoperative time, patients were transferred to the recovery room or to the wards when they recover from anesthesia. There was no difference in the usage of baricity of LA, volume of local anesthetics, gauge of spinal needle used, time of LA administered in CSF, and vertebral interspace drug administration between the two groups.

### 2.8. Data Collection Technique and Patients

The data structured questionnaire was prepared in English. The questionnaire was adapted from different articles with some modifications [14–17]. The data collection was done by four anesthetists and supervised by one anesthetist that has experience in research. The patients' sociodemographic data (age, sex, weight, height, BMI, ASA status, and duration of hypertension), type of procedure, duration of surgery, amount of blood loss, antihypertensive agent, total fluid infused intraoperative, and peak level of sensory block were recorded. Intraoperative blood loss was assessed from the drainage in suction bottles and checking the gauze and packs used and appropriate replacement was done. Baseline blood pressure (SBP and DBP) using noninvasive blood pressure (NIBP) and heart rate (HR) using pulse oximetry were measured in the 1^st^ one minute and later 2 minutes before spinal anesthesia was administered. After spinal anesthesia was performed, the above hemodynamic parameters were measured at 1, 3, 5, 10, 15, 20, 25, 30, 35, 40, 45, 50, 55, and 60 minutes. After that, the level of sensory block was evaluated with cold alcohol and pinprick after spinal anesthesia. The duration of surgery, fluid colloid, total fluid infusion at 1 hr, time of LA administered into the CSF (in seconds), and peak level of sensory block in each patient were noted. The data were labeled as group 1 for normotensives and group 2 for controlled hypertensive patients and were analyzed with statical software.

### 2.9. Data Quality Control

To ensure the quality of data, the following measures were undertaken. The questionnaire was adapted from different articles with some modification and checked by anesthesia professional experts' and advisors whether the adapted questionnaire measures the expected goal. After feedback was obtained from experts, the questionnaire was rewritten accordingly. A total of two days of training was provided to the data collectors and supervisors about the checklist recording. The training focused mainly on how to record properly each part of the questionnaire and how to approach the patient during data collection. Pretest of the questionnaire has been performed at Menelik II Referral Hospital with 5% of the total sample size (divided into two groups) which were not included in the actual study. The principal investigator and supervisor made daily supervision during the whole period of data collection. Every day, the questionnaires were reviewed and checked for completeness, clarity, and consistency by the supervisor and investigator. Incomplete data were not entered into a database prepared on Epi Info. Data cleanup and cross-checking were done before analysis on SPSS.

### 2.10. Statistical Methods

The data were checked manually for completeness and then entered and cleaned using Epi Info, version 7, and exported to Statistical Package for Social Sciences (SPSS) software, version 20. All continuous data were tested for normality by using Shapiro–Wilk's test with *p* > 0.05 and they were rechecked with a visual inspection of their histograms. We have also used Levene's test to check the homogeneity of variance (*p* > 0.05) for both N and CH groups.

Descriptive data were described in mean ± SD for normally distributed and median and interquartile range for nonnormally distributed data. Comparisons of numerical variables between the study groups, age, body weight, height, BMI and hemodynamic parameters, blood loss, and fluid management with respect to the normal distribution, were analyzed using an independent samples *t-*test. As the peak level of sensory block was nonnormally distributed, it was analyzed using Mann–Whitney *U-*test. Categorical variables between the study group's sex, occurrence of incidence of hypotension, and bradycardia were compared with Chi-square test. Statistical significance was determined at *p* value <0.05.

## 3. Results

### 3.1. Characteristics of Demographic Data and Comparison of the Groups

A total of 110 (55 controlled hypertension and 55 normotensive) patients who underwent surgery below the umbilicus under spinal anesthesia were included in this study. Both groups were comparable in terms of demographic and baseline hemodynamic parameters (Tables 1 and 2).

### 3.2. Comparison of Blood Loss, Intraoperative Fluid Management, and Duration of Surgery between the Two Groups

**Figure 1:**
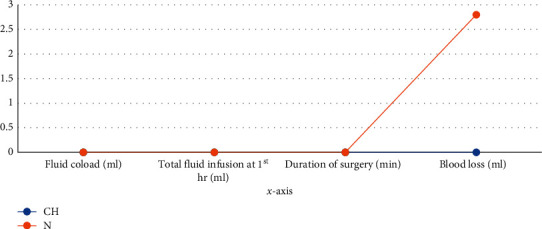
Variation in the fluid coload, total intraoperative fluid used, duration of surgery, and blood loss between the two groups at Black Lion Hospital from October 30, 2019, to January 30, 2020.

Fluid coload and total IV fluid used intraoperative, blood loss within the first one hour and duration of surgery did not differ statistically significantly between the two groups (*p* > 0.05). Both groups were comparable in terms of fluid coload, total IV fluid infused intraoperatively, blood lose, and duration of surgery at the first hour (Figure 1).

### 3.3. Comparison of Mean Systolic Blood Pressure

There was a statistically significant difference in mean systolic blood pressure value between the controlled hypertension (CH) and normotensive (N) group when compared with baseline in the same group at the measurement of 15^th^, 20^th^, 25^th^, and 30^th^ minutes with *p* value (*p*=0.025,  0.009,  0.009,  0.002, resp.).

The mean SBP was 132.8909 mmHg and 133.9455 mmHg in the CH group and N group, respectively, at baseline. The SBP was dropped in both groups after spinal anesthesia (Figure 1). The maximal fall of mean SBP was seen from 132.8909 mmHg to 116.5818 mmHg (12.3%) and 116.3455 (12.45%) mmHg at 25 min and 30 min in CH group and from 133.9455 mmHg to 122.0727 (8.9%), 122.3818 (8.6%), and 122.2364 (8.7%) mmHg at 35 min, 40 min, and 45 min in the N group, respectively. The increase in SBP following the maximal drop was seen to a greater degree in CH group than N group.

The insignificant result after 35 minutes may be that the hypotensive episodes could be effectively managed without any serious hazards to the patient.

### 3.4. Comparison of Mean Diastolic Blood Pressure

The mean diastolic blood pressure was comparable between the two groups. The mean DBP was 78.5455 mmHg and 79.0182 mmHg in CH and N group at baseline, before the patient administered spinal anesthesia. The mean DBP was dropped after spinal anesthesia and fall was maximally seen from 78.5455 mmHg to 73.8909 mmHg (5.9%) at 25 min in the CH group and from 79.0182 mmHg to 74.7455 mmHg (5.4%), 74.5455 mmHg (5.7%), and 74.5455 mmHg (5.7%) at 25^th^, 30^th^, and 35^th^ min was also dropped in the N group. The mean DBP increase following maximally drop was seen in the CH group and N group in postspinal anesthesia in the first hour.

### 3.5. Comparison of Mean Heart Rate

**Figure 2:**
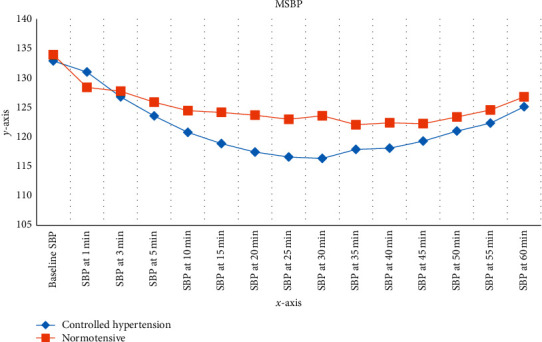
Variation in the systolic blood pressure (SBP) between two groups. The changes from baseline of the SBP in the first one hour after spinal anesthesia (*x*-axis duration and *y*-axis mean of SBP in mmHg) at Black Lion Hospital from October 30, 2019, to January 30, 2020.

As compared to the baseline, the mean record of heart rate in the controlled hypertension group was higher than baseline with the highest value to be 78.6364 mmHg and 79.4364 mmHg at the 1^st^ and 3^rd^ minutes. While in the normotensive group, all the mean record of HR was lower than baseline at each time interval with the first hour. The maximal drop of mean HR was from 78.2545 mmHg to 73.8909 mmHg (5.6%) at 35 min in the CH group and from 76.5636 mmHg to 72.7636 mmHg (4.96%) at 25 min in the N group. The increase in mean heart rate following maximal drop was seen to a greater degree in the CH group than the N group after spinal anesthesia (Figure 2).

### 3.6. Comparison of the Incidence of Hypotension and Bradycardia

The total number of patients that had a significant decrease of ≥25% of SBP from baseline was 13 (23.6%) and 4 (7.3%) in the CH and N group, respectively. There was a statistically significant *p* value of 0.018 showing that the incidence of hypotension was significantly seen in controlled hypertension patients who were regularly on antihypertensive medication and continued nifedipine on the day of surgery (Table 3).

The occurrence of bradycardia (HR < 60 b/min) was seen in 7 patients from each group and the rescue medication of atropine was injected in all cases. There was no statistically significant difference between the CH and N group on dropping the heart rate after spinal anesthesia (*p* > 0.05) (Table 3).

## 4. Discussion

The present study showed that, during spinal anesthesia, the incidence of hypotension occurrence in the controlled hypertension group was higher than the normotensive group. Hypotension, defined as systolic blood pressure, decreased by 25% and more than the baseline value [18]. This is the most significant predictor of morbidity and patient cardiac events. This study showed that the SBP had a drop of 25% more than the baseline which was seen in 13 (23.6%) in controlled hypertension and 4 (7.3%) in the normotensive group. This was a statistically significant difference between the two groups (*p*=0.018). This finding is consistent with the study done in India which was a comparison of hemodynamic response following spinal anesthesia between normotensive and controlled hypertension patients. In that study, the incidence of hypotension was found to be 8 (26.6%) in normotensive and 17 (56.6%) in controlled hypertensive patients with the same value defining a drop of SBP 25% and above from baseline, and there was a statistically significant difference between the two groups (*p*=0.01).

In similar prospective cohort studies, Rabbani et al. reported that the incidence of hypotension due to spinal anesthesia was 17 (34%) in normotensive and 31(62%) in controlled hypertension which was statistically significant with *p* value <0.05. This study was also consistent with our findings [13]. A possible explanation for the increased incidence of hypotension in controlled hypertension compared to the normotensive group could be in hypertensive patients increased sympathetic activity and norepinephrine level as well as decreased parasympathetic activity and persistent sympathetic stimulation. It may also cause loss of elasticity in the arterial wall and induce structural changes that in turn result in a decrement in blood pressure due to sympathetic blockade by spinal anesthesia and due to the continued nifedipine doses on the day of surgery [19].

In contrast to our results, Acar et al., showed that the incidence of hypotension between controlled hypertension and normotensive patients was not statistically significant (*p* > 0.05) [17]. The incidence of hypotension was 20% (6 out of 30) in controlled hypertension and 3.3% (1 out of 30) in the normotensive group. Another similar study, which was 1 or 2 decades older than ours, showing that the incidence of hypotension between controlled hypertension and normotensive patients was 55.5% and 43.8%, respectively, and this was also not statistically significant (*p* > 0.05) [14].

Overall, the current findings from our study did not match with the findings from the abovementioned two studies. The inconsistent result may be due to the different management protocols, techniques of patient monitoring, and antihypertensive medications used.

With regard to the change in heart rate in our study, the number of patients who developed bradycardia was 7 patients in each group, which is 12.7%, and there was no statistically significant difference between the two groups (*p* > 0.05). In those patients who developed bradycardia, rescue medications including atropine and vasopressors were administered. In another similar study (Dinakar et al.), it was shown that the occurrence of bradycardia was 4 (13.3%) patients in each group, which was not different. The variation in percentage between the current study and the other studies may be due to the difference in sample size used in those studies [17]. Another study which was done by Acar et al. showed the occurrence of bradycardia to be 6 (20%) in controlled hypertension and 7 (23.3%) in normotensive patients which was consistent with our findings with *p* value >0.05 [17].

The maximal drop of mean SBP was seen as 12.3% and 12.45% at 25 min and 30 min in the CH group and at 35 min, 8.9%; at 40 min, 8.6%; and at 45 min, 8.7% in the N group, respectively, where the drop of mean SBP in the CH group was higher than the normotensive group. Mean SBP between the two groups was statistically significantly seen at 15 min, 20 min, 25 min, and 30 min (*p*=0.025,  0.009,  0.009,  0.002).

A possible reason for nonsignificant after 30 min may be the episode of hypotension could be effectively managed without serious hazard to the patient **(**Figure 2).

**Figure 3:**
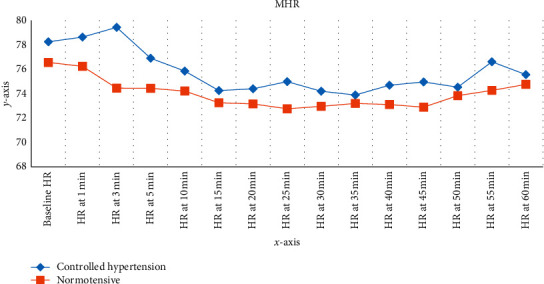
Variation in heart rate (HR) between two groups. The changes from baseline of the HR in the 1^st^ hour in postspinal anesthesia (*x*-axis duration and *y*-axis mean of heart rate in beat per minute) at Black Lion Hospital from October 30, 2019, to January 30, 2020.

The maximal fall of mean DBP was seen 5.9% at 25 min in the CH group and 5.4%, 5.7%, 5.7% in the N group at 25 min, 30 min, and 35 min, respectively, and there was no statistically significant difference between the two groups at all measurements over the period (*p* > 0.05) **(**Figure 3). Usually, diastolic blood pressure should remain unchanged or rise slightly. The exact DBP threshold has been widely debated or unclear. Furthermore, whether this threshold differs in patients with obstructive coronary disease who may be vulnerable to reduced coronary perfusion during diastole is a point of debate [20]. The peak level of sensory block was at the level of T10 (T6-10) for each group and there was no statically significant difference between CH and N patients.

### 4.1. Strength and Limitation of the Study

#### 4.1.1. Strength of the Study

The study participants were homogeneous between the two groups.

#### 4.1.2. Limitation and Scope of Further Research

There is a lack of current literature done in our country with a similar study design for comparison. The preoperative antihypertensive drugs were not protocolized properly and continued as before. This may have an impact on the blood pressure values. The blood loss was assessed from the drain output and the mops and swabs used. For appropriate assessment, quantitative measurement should have been done and the blood loss and fluid administration should have been compared. The definition of hypotension should be standardized and for this, a large multicentric trial may be undertaken in the future.

#### 4.1.3. Conclusion and Recommendation

Patients with controlled hypertension were more likely to develop hypotension than normotensive patients under spinal anesthesia with 0.5% hyperbaric bupivacaine, and there was a statistically significant difference in the incidence of hypotension occurrence between controlled hypertension and normotensive groups. The mean SBP between the two groups was statistically significant at 15 min, 20 min, 25 min, and 30 min; however, there was no statistically significant difference in mean diastolic pressure at all measurements over the period. In addition to that, there was no statistically significant difference in the occurrence of bradycardia under spinal anesthesia between the two groups. Based on the findings from this study, we recommend necessary precautions to be taken while administering spinal anesthesia in controlled hypertensive patients above the age of 40 as the higher incidence of hypotension can lead to deleterious cardiovascular effects, other end organ damage, and even sudden death in these patient groups. Hence, special attention should particularly be given to the preoperative optimization of patients, intraoperative hemodynamic and dermatomal level monitoring, appropriate positioning, and immediate administration of vasopressors in case of severe hypotension. In addition, this prompt administration of atropine should also be considered in patients who have developed bradycardia.

## Figures and Tables

**Table 1:** Comparison of demographic data and baseline hemodynamic parameter between the two groups: Black Lion hospital from October 30, 2019, to January 30, 2020.

Variables		CH (*n* = 55)	N (*n* = 55)	*p* value
Age in years (mean ± SD)^*∗*^		58.15 ± 10.662	60.78 ± 11.832	0.222
Sex	M (f (%))	40 (72.7%)	48 (87.3%)	0.095
	F (f (%))	15 (27.3%)	7 (12.7%)	
Weight in kg (mean ± SD)^*∗*^		66.4364 ± 5.88395	65.2727 ± 7.10361	0.352
Height in cm (mean ± SD)^*∗*^		167.0364 ± 3.91561	166.4909 ± 4.96235	0.524
BMI in kg (mean ± SD)^*∗*^		23.9244 ± 2.55949	23.5787 ± 2.58013	0.482
Baseline SBP (mmHg)^*∗*^		132.8909 ± 4.57721	133.9455 ± 5.66803	0.285
Baseline DBP (mmHg)^*∗*^		78.5455 ± 7.726380	79.0182 ± 7.80430	0.75
Baseline HR (bpm)^*∗*^		78.2545 ± 9.3238400	76.5636 ± 10.34903	0.37

Data are given as (mean ± SD).^*∗*^Independent samples *t*-test^*∗*^ (median (IQR)). f: frequency.

**Table 2:** Comparison of the peak level of sensory block and preoperative medication use between the two groups: at Black Lion Hospital from October 30, 2019, to January 30, 2020.

Variables	CH (*n* = 55)	N (*n* = 55)	*p* value
	Mean ± SD	Mean ± SD	
Peak level of sensory block^*∗∗*^	T10 (T6-T10)	T10 (T6-T10)	0.557
Antihypertensive medication (n)			
Calcium channel blockers	33	Nil	
Diuretics	4	Nil	
ACI inhibitors	15	Nil	
ACI inhibitors and beta-blockers	3	Nil	
Nifedipine morning dose (mg)	20–30	Nil	

Morning antianxiety (diazepam)	0.15–0.2 mg/kg	Nil	
Time of LA administered into CSF (in seconds)^*∗*^	16.7273 ± 2.3994900	16.4545 ± 2.29184	0.543

^*∗*^Independent samples *t*-test. ^*∗∗*^Mann–Whitney *U*-test.

**Table 3:** Comparison of the incidence of hypotension, bradycardia, vasopressor consumption, and atropine usage in response to bradycardia between the two groups: at Black Lion Hospital from October 30, 2019, to January 30, 2020.

Parameter	CH (*n* = 55)	N (*n* = 55)	*p* value
SBP within ≥25% drop *n* (%) (hypotension)	13 (23.6%)	4 (7.3%)	0.018
Bradycardia *n* (%)	7 (12.72%)	7 (12.72%)	>0.05 (Ns)
Atropine *n* (%)	7 (12.72%)	7 (12.72%)	>0.05 (Ns)

Chi-square test with *p* < 0.05 considered statistically significant, Ns: nonsignificant, SBP: systolic blood pressure.

## Data Availability

All the data are available within the manuscript. The datasets used and/or analysed during the current study are available from the corresponding author based on reasonable request.

## Abbreviations

ASA: American Society of Anesthesiologists
BP: Blood pressure
HR: Heart rate
MAP: Mean arterial pressure
CH: Controlled hypertension
BMI: Body mass index
CSF: Cerebrospinal fluid
LAs: Local anesthetics
mmHg: Millimeter mercury
SA: Spinal anesthesia
L3-L4: Lumbar vertebrae 3 to 4
T5: Thoracic vertebrae five
CCB: Calcium channel blockers.

## Additional Points

Operational definitions:

- Hypotension: SBP ≥25% decrease from the baseline value of the SBP (13, 24).
- Controlled hypertensive: according to the National Institute of Health Public, the patient is treated and optimized for ≥2 weeks and clinically BP getting <140/90 mmHg as a result of treatment by antihypertensive medication/s (25).
- Normotensive: according to the American Medical Association, who had not been diagnosed having a history of hypertension and previously not exposed to antihypertensive medication and for the age of <60 years old as the clinical BP getting <140/90 mmHg and adults aged 60 years and above the clinical BP getting <150/90 mmHg (29).
- Bradycardia: HR < 60 beats per minute.
- The level of sensory block: loss of sensation for cold water or alcohol and pinprick will be recorded bilaterally in the anterior axillary line or midclavicle line.
- Autonomic block: blocking of the sympathetic nervous system and with their dominating parasympathetic nervous system.
- Baseline hemodynamic: measuring of blood pressure and heart rate before performing spinal anesthesia.
